# Evaluation of sliding baseline methods for spatial estimation for cluster detection in the biosurveillance system

**DOI:** 10.1186/1476-072X-8-45

**Published:** 2009-07-17

**Authors:** Jian Xing, Howard Burkom, Linda Moniz, James Edgerton, Michael Leuze, Jerome Tokars

**Affiliations:** 1 Centers for Disease Control and Prevention, 1600 Clifton Road NE, Atlanta, GA 30333, USA; 2 The Johns Hopkins University Applied Physics Laboratory, 11100 Johns Hopkins Road, Laurel MD 20723, USA; 3 Edge Space Systems, Inc., PO Box 310, Glenelg, MD 21737, USA; 4 Oak Ridge National Laboratory Oak Ridge National Laboratory, PO Box 2008, MS 6367, Oak Ridge, TN 37831, USA

## Abstract

**Background:**

The Centers for Disease Control and Prevention's (CDC's) BioSense system provides near-real time situational awareness for public health monitoring through analysis of electronic health data. Determination of anomalous spatial and temporal disease clusters is a crucial part of the daily disease monitoring task. Our study focused on finding useful anomalies at manageable alert rates according to available BioSense data history.

**Methods:**

The study dataset included more than 3 years of daily counts of military outpatient clinic visits for respiratory and rash syndrome groupings. We applied four spatial estimation methods in implementations of space-time scan statistics cross-checked in Matlab and C. We compared the utility of these methods according to the resultant background cluster rate (a false alarm surrogate) and sensitivity to injected cluster signals. The comparison runs used a spatial resolution based on the facility zip code in the patient record and a finer resolution based on the residence zip code.

**Results:**

Simple estimation methods that account for day-of-week (DOW) data patterns yielded a clear advantage both in background cluster rate and in signal sensitivity. A 28-day baseline gave the most robust results for this estimation; the preferred baseline is long enough to remove daily fluctuations but short enough to reflect recent disease trends and data representation. Background cluster rates were lower for the rash syndrome counts than for the respiratory counts, likely because of seasonality and the large scale of the respiratory counts.

**Conclusion:**

The spatial estimation method should be chosen according to characteristics of the selected data streams. In this dataset with strong day-of-week effects, the overall best detection performance was achieved using subregion averages over a 28-day baseline stratified by weekday or weekend/holiday behavior. Changing the estimation method for particular scenarios involving different spatial resolution or other syndromes can yield further improvement.

## Background

### The problem of detecting anomalous case clusters

Epidemiologists responsible for routine public health surveillance seek early indications of possible disease outbreaks. A growing collection of data types has become available to aid this surveillance, but with the analysis of these data comes the need to reduce the search possibilities. With cluster detection methodology, analysis of data sources can help by indicating the likely location and extent of a potential outbreak. Spatial and spatiotemporal scan statistics have the additional advantages of controlling the number of alarms resulting from multiple testing and accounting for usual clustering patterns, as found in the data [1].

Some natural clustering is expected, as in urban areas with high population density. If cluster detection methods do not account for this heterogeneity, customarily high concentrations will be signaled as anomalous. Only departures from the usual distribution should be flagged. In the implementation of the widely used SaTScan software [2], a detection statistic is applied to all candidate clusters, and the cluster with the highest statistical value is tested for significance. The statistic is a function of observed and expected values both inside and outside the candidate cluster. Thus, an accurate estimate of the expected spatial distribution is necessary for determination of relevant clusters with high sensitivity at reasonable false alarm rates. This requirement is consistent whether the detection approach uses scan statistics or another methodology.

A natural choice for the expected spatial distribution is the census population by sub-region of the geographic area under surveillance. In the United States, for example, population counts stratified by 3-digit zip code, 5-digit zip code, or census tract are freely downloadable [3]. This choice is appropriate for some applications of scan statistics to chronic disease surveillance where the data give true population rates relative to census totals and long-term effects are of interest. One problem with using these counts is that they represent the population measured at the most recent census, taken only every 10 years, so they do not include more recent population shifts. A more serious problem is that some biosurveillance data sources are not representative of the entire population, so they might produce expectations that are biased relative to the data of interest. This bias typically occurs because the data represent only one segment of the population that is not evenly dispersed over the region. Examples are data covering only military personnel and their dependents, as in this study, participants in a health maintenance organization or other medical plan, or customers of a pharmacy chain. Organizations furnishing such data typically do not or cannot furnish details of their spatial coverage. Therefore, system developers have used past data to form spatial expectations.

If substantial (typically multi-year) quality historical data are available, detailed modeling of counts at the sub-region level could be attempted to calculate expected distributed counts. Kleinman et al [4] obtained significantly reduced false alarm rates with this approach. However, health monitors typically need to apply data with little data history, and longer data history is likely to be unrepresentative of current spatial patterns because of changes in population behavior, in participation of data providers, and in data collection systems. This article focuses on data-based methods of computing expected count distributions assuming that only 1–8 weeks of recent data are available.

For clarity, we distinguish two separate problems in applying data with spatial information to detection of disease clusters. The first problem is how to aggregate the data into sub-regions. Ozonoff et al and Olson et al. [5,6] demonstrated that the level of aggregation can strongly affect the power of scan statistics for cluster detection. The second problem is that given the total number of cases occurring in a monitored region and the chosen partition of sub-regions, what is the expected distribution of cases among these sub-regions *if no public health event of interest is underway*? Reasonable agreement with this distribution might be seen as the null hypothesis that is rejected if a case cluster is found at a level of significance above a preset threshold. Practical data considerations often drive the answer to the first problem. Privacy laws typically preclude the use of patient addresses or other exact location fields, so zip codes, census tracts, and locations of clinics specified in data records have been used. The second problem is the subject of this article.

### Study dataset

The choice of an effective method for estimating the case distribution is a function of the monitored data source. Even within the same data source, for example, estimation of the current distribution for a broad, seasonal syndromic filtering of clinical records is likely different from the same estimation for a rare sub-syndrome occurring evenly throughout the year. For the current study, we used data from BioSense, a US national biosurveillance system operated by the Centers for Disease Control and Prevention (CDC) [7]. This system uses computer databases for automated disease surveillance. The source of data for the study was the BioSense collection of US Department of Defense (DoD) military outpatient clinic data records. Since 2004, these data records have been sent daily to CDC through the TriCare Management Activity, which manages clinical patient data for all US military treatment facilities. The patient base includes personnel on active duty, retirees, and dependents. Patient record fields provide key information including the visit date, age, gender, residence and treatment clinic zip codes, and for medical classification, a code from the *International Classification of Diseases, Ninth Revision *(commonly called *ICD-9*), used by hospital and physicians' office personnel to report billing information to insurance companies [8].

**Figure 1:**
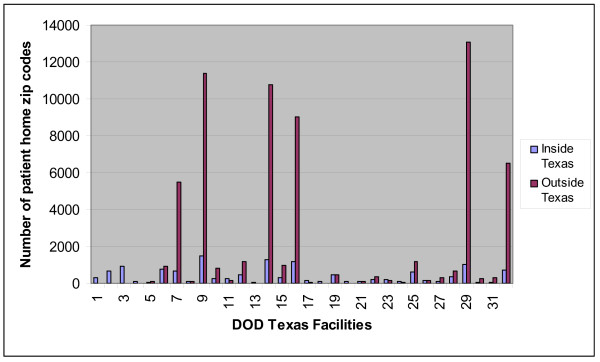
**Number of patient home zip codes inside and outside Texas per DoD Texas facility**.

The study dataset included 3 years (1/1/2004–12/31/2006) of DoD patient records from military outpatient clinics in the state of Texas, which has a large, widespread military population. These records were classified according to their *ICD-9 *code fields into 11 standard BioSense syndrome groups [9]. The respiratory and rash syndromes have been chosen for this study to represent high-count, seasonal data behavior and sparse, nonseasonal behavior, respectively. The record fields allowing spatial aggregation were the zip code of the clinic where the patient was seen and the zip code of patient residence. The patient residence zip codes were distributed nationwide, mostly outside of Texas. Figure 1 illustrates the number of records with residence zip codes inside and outside Texas for each DOD facility in Texas.

One obvious difference between the geographic patient distribution for the study dataset and the general census distribution results from the large fraction of non-Texas residents among these patients who live near the military bases, with the result that zip codes near those bases are overrepresented relative to census figures. However, for reasons of both patient privacy and military operational security, the spatial distribution of people eligible for treatment at base clinics is unavailable for analysis. Because of commuting by Texas residents, it cannot be assumed from Figure 1 that nearly all patient residences are close to treatment facilities. Many commuters are eligible for treatment at military clinics throughout the continental United States, and in western states such as Texas, commuting distances of over 50 miles are common. For these reasons, data-based spatial estimation methods are required. The Methods section discusses the data preparation and the estimation methods tested for cluster detection.

## Methods

### Treatment of data record spatial information

Using location fields in patient records to seek infectious disease clusters, the underlying assumption is that the field value approximates the location of exposure to the disease-causing agent. The location of exposure is difficult to determine in individual-based studies and much more so in automated, population-based systems. The records in the study dataset contain fields for zip codes of the treatment facility and of the patient's home. In the DoD population whose records compose this dataset, most of the active duty personnel work on military bases that include treatment facilities. Therefore, a working hypothesis is that the facility zip code should be used to detect an outbreak in which exposures occur in the work environment; whereas, the home zip code should be used to detect an outbreak resulting from residence-based exposures. However, the patient home zip codes in the study data often refer to the home town of the patient, not to the zip code of military duty at the time of treatment. In such cases, the facility zip code is a better choice for the current residence. There is no practical way to know which zip code to use for the residence-based analysis, so in view of the number of patients who commute, we have used the following "100-mile rule" on patient zip codes to prepare the data before the analysis.

▪ First, we included all records that have either a facility zip code or a home zip code inside Texas. We also included records from four facilities outside of Texas but near the Texas-Oklahoma and Texas-Louisiana borders because of the large numbers of Texas residents commuting across the border and using those facilities.

▪ Second, we calculated for each record the distance between the home and facility zip codes.

▪ For the residence-based analysis, we used the patient home zip code if this distance was less than 100 miles. Otherwise, we used the facility zip code.

▪ If the home zip code field was empty, the facility zip code was used as the residence zip code. If the facility zip code was missing or outside Texas, the home zip code was used.

We used this reasonably simple rule because a more detailed assignment would require information that was available neither for the study nor for routine monitoring. In a normal situation most people would probably not travel more than a couple of hours to a facility unless there were no other choices.

**Figure 2:**
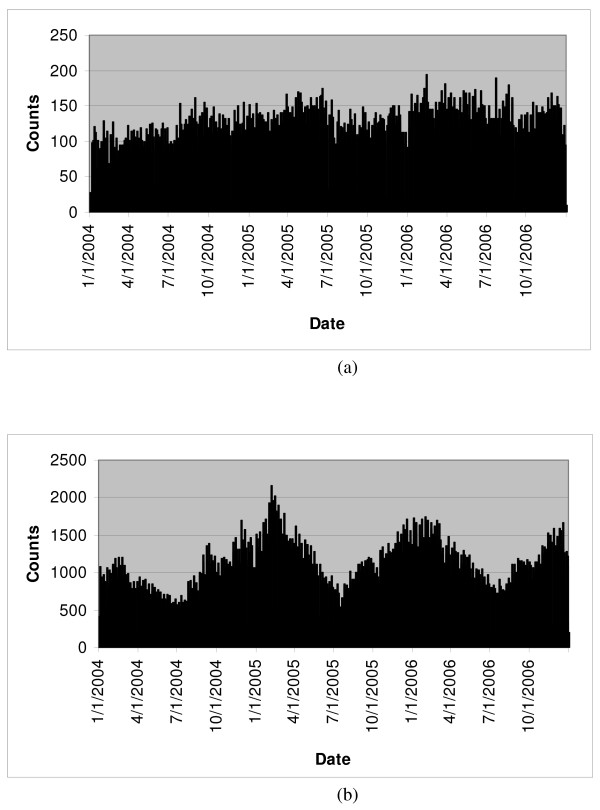
**Distribution of syndrome counts by date**. **(a) **for rash, **(b) **for respiratory.

**Figure 3:**
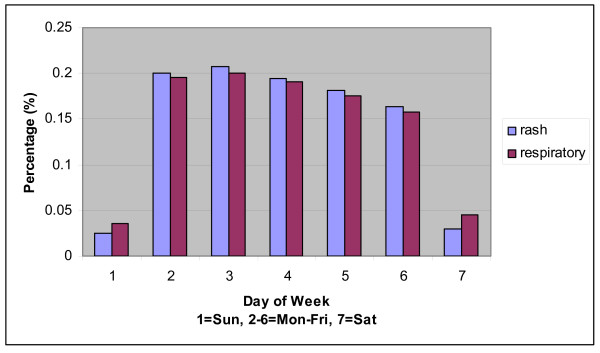
**Percentage of counts by day of week**.

Restriction of the study data set to those records classified in syndrome groups yielded 1,233 residence zip codes and 32 facility zip codes. The residence zip codes were well distributed in the eastern half of the state with high concentrations around the cities, but much sparser to the west. Most of the 32 facility zip codes were to the east with a high concentration in the San Antonio area. The west was covered only by two facilities at El Paso in the western tip. The mean daily record counts per residence zip code were 0.29 and 2.67 for rash and respiratory syndromes, respectively. The corresponding mean daily record counts per facility zip code were 4 and 38. Thus, respiratory counts exceeded rash counts by a factor of >9. Although rash counts have a relatively smooth pattern with a slightly increasing trend during the 3-year period, respiratory counts have a strong seasonal pattern (Figure 2). Both syndromes have a strong day-of-week effect (Figure 3).

### Calculation of expected counts

**Figure 4:**
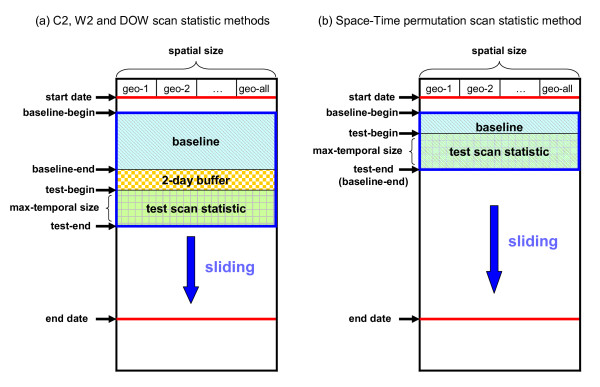
**C2, W2, DOW and Space-Time permutation scan statistic methods**.

We used two types of estimation approaches to calculate expected counts: a baseline-mean approach and a space-time permutation approach. Figure 4 illustrates these two approaches. In each half of this figure, columns geo-1, geo-2 etc. are used to indicate subregions. Time is shown vertically, with a set of rows for each block of days. The "start date" and "end date" give the study date limits including multiple runs. For a given run in the series, "baseline-begin" and "baseline-end" show the data dates bounding the baseline, followed by a 2-day buffer and then by the dates whose data are being tested for clusters. After each run, the arrows indicate that these blocks move forward by a day.

#### Baseline-mean approach

Figure 4(a) shows the baseline-mean approach, referring to a sliding, fixed-length baseline period of days before the current day. We describe three methods depending on how the baseline days are used to estimate the current day count.

▪ ***Unstratified mean*, or *C2-based***: The expected count for a sub-region was computed as the mean count over all baseline days for that sub-region. For the 7-day baseline, this method gives the same expected count for each zip code as that used for the temporal Early Aberration Reporting System (EARS) C2 algorithm [10].

▪ ***Weekday/weekend mean*, or *W2-based***: The expected count for a zip code is over the only weekday (weekend) baseline day if the current day is a weekday (weekend). Days occurring on calendar holidays are classified with weekends because in several BioSense datasets, holiday counts are similar to weekend counts.

▪ ***Individual weekday mean*, or *day-of-week (DOW) based***: The expected count for a zip code is more than only baseline days on the same day of the week as the current day. Days occurring on calendar holidays are classified with Sundays.

We used baseline lengths of 7, 28, and 56 days (1, 4, and 8 weeks) with a 2-day buffer between baseline and test day. As in the EARS methods [10], the purpose of the buffer is to reduce the effect of any current outbreak on the baseline data. We excluded the 7-day baseline from W2-based and DOW-based methods because the 7-day baseline is too short for weekends and holidays. For each test day, these averages were calculated and conditioned on the daily case total to obtain expected counts for the scan statistics (see Appendix).

The expected count for each sub-region is calculated as a fraction of the total number of cases in the test period, set to 1 day in this study. It is critical to correctly estimate this fraction to the correct recognition of spatial aberrations. In the traditional scan statistics application of SaTScan, this fraction is the population of the sub-region divided by the total population of the surveillance region. In the current data environment, where the denominator population is unknown, we estimate this fraction as the number of sub-region baseline cases divided by the sum of baseline cases in the entire region. The only difference in this estimate for the C2-, W2-, and DOW-based prediction methods is which days are included in the baseline.

This approach must be adjusted for the possibility that the expected count for a sub-region might be zero. For many test statistics, including the Poisson generalized likelihood ratio [11] used here, the ratio of observed counts *Obs*_*j *_to expected counts *Exp*_*j *_cannot be zero for any sub-region *j *because the ratio *Obs*_*j*_/*Exp*_*j *_is used in the statistic. The space-time permutation method [11], sketched in the next section, avoids this problem at the cost of including counts from the test period in the baseline with no buffer interval. Including the test period guarantees that any sub-region with a positive number of observed cases will have a positive number of baseline cases. A second approach used here is to add 1 to the baseline sum for each sub-region. We give an example here for estimating the case count for sub-region *j *by the W2-based method, or weekday/weekend stratification, if one or more of the following is true:

a) The test day is a weekday.

b) There are 50 cases in the entire region for the test day and 200 data subregions.

c) There are a total of 1,000 cases on weekdays in the baseline.

d) Sub-region *j *has 75 cases on weekdays in the baseline.

If one or more of the cases above are true, then the expected number of cases is [(75 + 1)/(1000 + 200)] * 50 = 3.167. See Appendix 1 for the formal statement of this estimation method.

##### (1) Space-Time permutation approach

Figure 4(b) shows the space-time permutation approach. In this approach we get estimated counts by conditioning on space-time marginal (SPM) totals for both geo-coordinates and days as in the SaTScan space-time permutation option [11]. In other words, expectations are based on keeping the total counts constant for both individual days for all subregions, and individual subregions for all days. For each randomization trial, the set of dates for all records is kept fixed and the set of subregions shuffled to keep these marginal totals constant. For a purely spatial scan statistic, this method is equivalent to the unstratified mean method with the baseline including the test period. The baseline might also be restricted as in the W2-based and DOW-based methods. As noted above, this approach guarantees that the baseline sum will be positive for any sub-region with positive count in the test period, but the disadvantage is that including the test day biases the baseline distribution.

### Software implementation

We implemented the algorithms described in the previous sections into C and MatLab computer programs both for research purposes and for possible use in BioSense. Development of codes giving identical random draws in both software environments allowed perfect agreement of results despite the diverse coding methods. These methods were also developed to facilitate comparison of the spatial estimation methods, to allow inspection of all variables at all stages, and to incorporate two of the following features: (1) computation of the statistical significance of maximum likelihood clusters using the extreme value (or Gumbel) distribution [12], and (2) for the capability to inject realistic signals on the background data for detection performance testing. The C program runs in PC and/or Unix platforms and is callable from SAS code. This code does not have all of the features of SaTScan. Readers interested in obtaining the code should contact the corresponding author.

### Cluster search space

Our implementation searches cylinders with circular bases. The cylinder height is the number of days in the current test period, so that for a 7-day cylinder, a candidate cluster might have from 1 to 7 days of data. The analysis that follows focuses on comparing spatial estimation methods and is restricted to 1-day cylinders. For the spatial search, any point grid of possible cluster centers could be used, and our study used the set of centroids of zip codes in the data. For analyses based on facility and presumed residence zip codes, the numbers of zip codes were 32 and 1,233, respectively. We defined the radius of a candidate cluster as the maximum distance from the center to another centroid in the cluster, and we allowed radii as large as 100 km for the residence zip code runs and 300 km for the clinic zip runs, based on possible care seeking behavior.

### Test statistic

We applied the *Poisson Generalized Likelihood Ratio*, *GLR *[11]. Presume that on test day t, there are *Obs *cases observed within a candidate cluster, *Exp *cases expected (calculated by one of the methods of this paper or otherwise modelled) in the cluster, and *Tot *is the total number of cases in the entire surveillance region since the beginning of the baseline. Our implementation followed the common practice of restricting attention to clusters for which more cases are observed than expected. When this condition fails, we set the test statistic to zero, and when Obs > *Exp*, the test statistic is GLR, or the logarithm of:



This statistic gives a measure of the likelihood of the observed counts inside and outside the cluster given the expected distribution and the total number of observed cases. Candidate clusters within the search space are ranked in descending order by their GLR values. The next step is to decide which if any of these clusters should be flagged as statistically significant.

### Significance testing

The LLR values used as the test statistic do not comply with a known distribution in general, so thresholds for significance are usually determined empirically. The empirical approach is to randomly generate a large set of simulated data distributions under the null hypothesis of the expected spatial distribution for the test period. For each simulated distribution *k*, apply the same search procedure used for the observed data to obtain the maximum test statistics *max(LLR(k)) *over all candidate clusters. Derive the LLR threshold for significance from the set of *max(LLR(k)) *and apply it to decide if one or more of the clusters found using the observed data are significant. Specific steps include:

#### Step 1

We use the same procedure for generating null distributions for the C2-based, W2-based, and DOW-based estimation methods. In this procedure, we form a probability vector



from the set of sub-region expected values *E(j) *derived by the estimation method of choice, and *J *is the number of sub-regions. Let *N *be the total number of cases observed on a day in which data are analyzed. For each of these cases, we choose a sub-region with a multinomial random draw using {*p*(*j*), *j *= 1,,, *J*}. A random number *x *between 0 and 1 is drawn using the uniform distribution, and sub-region *j *is chosen if *x *is between ∑^*j-1*^*p(j) *and ∑^*j*^*p(j)*, so that the likelihood of choosing region *j *is proportional to *p(j)*. We thus obtain a random distribution corresponding to the expected ratios. For seeking space-time clusters in test periods of more than one day, we apply this same procedure for each test day.

For the space-time permutation method, we generated trial distributions by implementing the procedure in [11], keeping the dates of all baseline cases fixed and shuffling their sub-regions. As noted above, the baseline includes the test period in this method, so this shuffling gives a new test distribution. For efficiency, we achieved this randomization using the Fisher-Yates Shuffle, commonly called the Knuth Shuffle [13].

#### Step 2

We applied the search for the maximum test statistic for each random trial distribution exactly as for the observed data and collected the set of trial LLR maxima.

#### Step 3

To determine significance of the observed LLR maxima, we applied the Gumbel distribution using parameters derived from the set of trial maxima [12]. The procedure is to derive Gumbel parameters *a *and *b *from these maxima [14] and then compute a p-value p* for cluster test statistic LLR* using:



The p-value is then tested for significance against a threshold chosen to control the background cluster rate. We used a p-value threshold of 0.0027, for a nominal daily background rate of one cluster per year, in the results below. Following [12], this significance method has two advantages over simply ranking LLR* among the trial maxima. First, fewer trial distributions are required to obtain a useful p-value estimate, and second, the Gumbel p-values are on a continuous scale so that strong significance can be inferred without a huge number of trials.

### Detection performance assessment

Reliable quantitative measurement of the sensitivity and specificity of a cluster detection method requires multiple signals distributed in time over the sub-regions of interest. Documentation of authentic outbreaks sufficient to provide enough detail for this purpose is very rare. Previous power studies [15,16] aimed at chronic disease applications focused on increased risk in a few sub-regions. However, infectious disease surveillance is concerned with detection of transient events in which cases are variably distributed in the region of interest. For a controlled, quantifiable detection performance capability, we therefore added to the software an injection component that allows the user the capability to: (1) specify the injected signal in detail, including the number of cases to inject, the epidemic curve of these injected cases by day, and the geometry of distribution of these cases; (2) produce the injected cases for each day using random draws and add them to the background data; (3) run the scan statistics for a set of days including the days of injected cases to determine whether clusters are detected at critical significance levels for the injected signals; and, (4) conduct sets of repeated trials of these tests at different start dates to allow computation of detection sensitivity and timeliness. The stochastic generation of signals and changing data background over time provides realistic variability for these trials.

The signal generation method is as follows:

▪ For each trial, a signal start date is selected, and we generate a stochastic epidemic curve using random draws from a lognormal distribution [17,18].

▪ Parameters of this distribution are supplied by the user to control the median peak day and time spread of the injected cases.

▪ These random draws are tabulated to give the total number of injected cases on each day. For each day with injected cases, these cases must be assigned to sub-regions, or zip codes in this study.

▪ Three geometries have been implemented to reflect hypotheses of spread of an aerosol pathogen. The implemented geometries include radial, wedge-shaped, and hourglass-shaped spread at rates that the user may specify.

▪ For the results given here, we used the radial spread, in which the drop-off of cases from the centre zip code is proportional to *e*^-*d*/*k*^, for a dispersion factor *k *that determines the radial decay rate.

In the current study, total signals consisted of either 50 or 100 cases added to the original respiratory counts for either home or clinic zip codes. For epicurve generation, we used lognormal parameters ζ = 1.3, σ = .4. These parameters gave region-wide time series epidemic curves of 8–13 days in duration. The radial spatial distribution was calculated using distance from the signal centre in kilometres, and for our Texas DoD dataset, we used a dispersion factor of *k *= 18 for a plausible spread of cases. We modified this spatial distribution procedure to avoid producing obvious signals by injecting large numbers of counts in zip codes where there were few or no baseline counts. For this reason, the maximum allowed signal for each zip code for each day was at least 2, but no more than the maximum number of background cases in that zip code for the case day of week. We ran the distribution algorithm imposing this criterion recursively until all cases were distributed. For the repeated trials to measure detection sensitivity, we chose a start day for the first signal, and in each subsequent trial, the start date was advanced by 8 days to sample the background counts at different seasons, avoid day-of-week bias, and reduce correlation among successive trials. We generated these trials over approximately 3 years of background data to obtain 127 trials. For comparison of detection capability using residence zip codes and facility zip codes, we first produced injected signals at the resolution of the 1,233 residence zip codes and ran the scan statistics at that resolution. We then distributed these cases to the 32 facilities using distributions based on the distance to each facility, for comparison runs at the coarser resolution.

For the sequential runs without injects to assess background cluster rates, the update procedure is depicted in Figure 4. Our primary purpose was to compare spatial estimation methods, so the test period for all runs was a single day with no look-back, the purely spatial case. For dates 1/1/2004 to 12/31/2006, we started the first run at the end of the 2-day buffer after the first baseline period. The maximum spatial cluster sizes were 300 km and 50 km for DoD facility zip scanning and patient zip scanning, respectively.

## Results

Cross-checking results of the C and Matlab software described above for implementing scan statistics, we compared the four estimation methods described in the Methods section using both facility and patient residence zip codes with 7-, 28-, and 56-day sliding baselines. The measures of effectiveness were compared using the background cluster rate and sensitivity to simulated clusters at chosen significance thresholds.

### Results of background cluster rate comparisons (a false alarm rate surrogate)

We applied the four estimation methods to scan all test dates and all coordinates in this study dataset. Because they are to be used for daily surveillance, and disease outbreaks are expected to be rare, a primary concern for using these methods is the actual cluster determination rate, or how often the method will indicate a need for investigation. If alarms occur too often, then the alarms corresponding to true health events might be ignored.

We ran the purely spatial cluster detection algorithm for the 3 years of data with no correction for temporal multiple testing to get the relative cluster determination rates of the four methods. In the absence of information about any true outbreaks during the data period, we could not refer to significant clusters as true or false alarms, so as a surrogate for a false alarm rate, we measured the background alarm rate as the number of significant clusters per unit time for the p-value 0.003.

**Figure 5:**
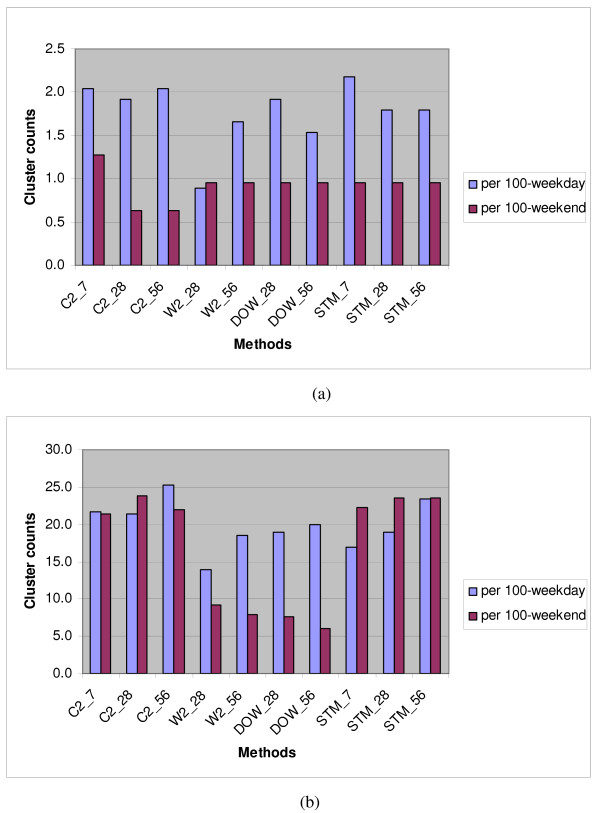
**Syndrome mean cluster counts per 100-weekday versus 100-weekend identified with DoD facility zip code**. **(a) **for Rash, **(b) **for respiratory.

**Figure 6:**
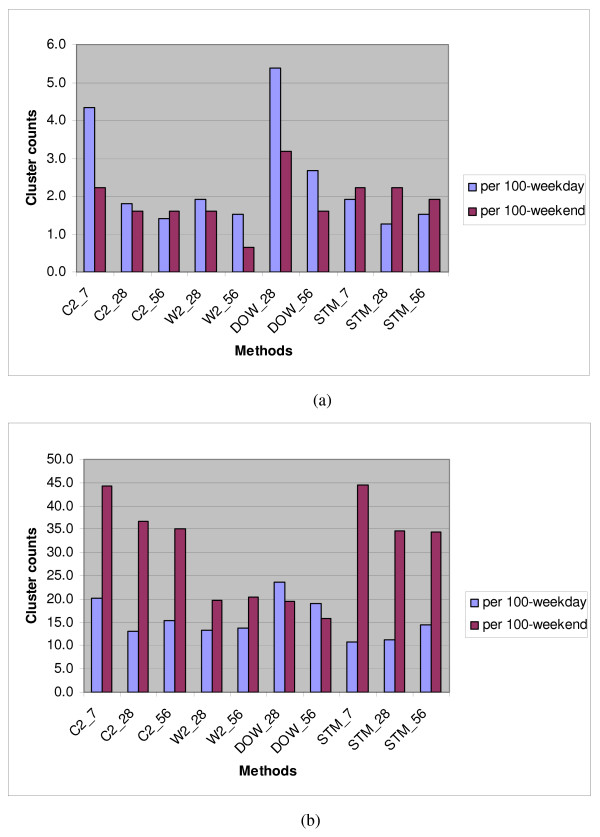
**Syndrome mean cluster counts per 100-weekday versus 100-weekend identified with patient residence zip code**. **(a) **for rash, **(b) **for respiratory.

Table 1 shows the number of significant clusters found per 100 weekdays and per 100 weekends, counting as significant those clusters with at least 2 sub-regions per cluster and with Gumbel p-value ≤ 0.003. Figure 5 shows the background cluster rates using the facility-level zip codes for (a) the rash syndrome and (b) the respiratory syndrome. Analogous plots for runs using residence-level zip codes are given in Figures 6(a) and 6(b). Key observations are:

**Table 1:** Number of clusters found per 100 weekdays/weekends, DoD Texas, 2004–2006, (p-value = 0.003, sub-region = 2).

**Spatial Resolution**	**Syndrome**	**Weekday/Weekend**	**Method/Baseline Duration (days)**									
			
			**C2**			**W2**		**DOW**		**STM**		
			
			**7**	**28**	**56**	**28**	**56**	**28**	**56**	**7**	**28**	**56**
**Facility Zip**	**Rash**	**Weekday**	2.0	1.9	2.0	0.9	1.7	1.9	1.5	2.2	1.8	1.8
		
		**Weekend**	1.3	0.6	0.6	1.0	1.0	1.0	1.0	1.0	1.0	1.0
	
	**Respiratory**	**Weekday**	21.6	21.4	25.3	13.9	18.5	18.9	19.9	17.0	18.9	23.4
		
		**Weekend**	21.3	23.9	22.0	9.2	8.0	7.6	6.1	22.3	23.6	23.6

**Residence Zip**	**Rash**	**Weekday**	4.3	1.8	1.4	1.9	1.5	5.4	2.7	1.9	1.3	1.5
		
		**Weekend**	2.2	1.6	1.6	1.6	0.6	3.2	1.6	2.2	2.2	1.9
	
	**Respiratory**	**Weekday**	20.1	13.0	15.3	13.3	13.7	23.7	18.9	10.9	11.1	14.5
		
		**Weekend**	44.3	36.6	35.0	19.7	20.4	19.4	15.9	44.6	34.7	34.4

▪ From Table 1, in general, the rash syndrome yield lower cluster rates than the respiratory syndrome, probably because of the seasonality and high mean and overdispersion of the respiratory counts.

▪ For the rash syndrome, the W2 estimation method with the 28-day baseline has a clear advantage over the unstratified averaging of the C2 method at the facility level, but not at the residence level, at which the day-of-week effect is less clear. The plotted levels of cluster alerts suggest that the full respiratory syndrome is too noisy for this dataset in the sense that its high mean and overdispersed variance cause much more significant but random clustering than would be expected for a sparser outcome variable, or especially for a dataset in which spatial distribution is similar to that of census data. Replacement by a more specific sub-syndrome such as influenza-like illness (ILI) should be considered; even though ILI counts are also seasonal, counts are much smaller, and their spatial distribution is likely to be fairly stable for most of the year.

▪ For the respiratory syndrome, management of day-of-week (DOW) effects is a primary concern, and the W2 and DOW methods clearly yield fewer cluster alerts than methods that ignore these effects. See Figures 5(b) and 6(b).

▪ The longest baseline of 56 days gives reduced background clustering only in certain circumstances, such as when the DOW method is used at the residence zip level. The finer temporal DOW stratification, combined with the finer spatial stratification, appears to require a longer baseline. For coarser aggregation, the longer baseline can actually increase the number of cluster alerts, likely because of seasonal effects.

▪ Extending the temporal stratification from the W2 to the DOW method never yields a clear overall advantage in reduced alerting.

▪ The W2 method with a 28-day baseline is a safe choice for controlling the number of cluster alerts overall and gives as clear advantage in some situations. However, for a sparse outcome variable such as the rash visit counts and a fine spatial subdivision of records, a longer baseline might reduce the alerting.

### Sensitivity comparison with signal injection

As described in the Methods section, we randomly generated 127 visit count signals with a region-wide lognormal epidemic curve and a radically decaying spatial distribution. Separate signal sets were generated for totals of 50 and 100 outbreak cases. For each set, we added these signals to the authentic respiratory syndrome background data for a series of detection trials.

**Figure 7:**
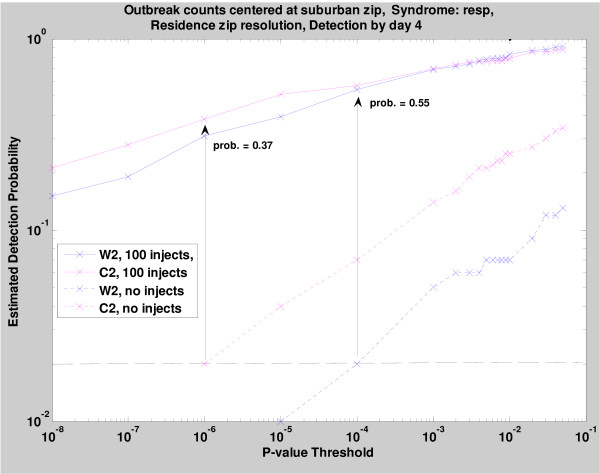
**Sensitivity versus p-value threshold stratified by C2 and W2 detection methods associated with no injects (background) and 100 injects**.

Figure 7 shows the sensitivity versus p-value threshold for the C2 and W2 detection methods associated with no injects (background) and 100 injects. When no injects were used, C2 had a higher background cluster rates than W2 for a given p-value. A background alert rate of 0.02 (indicated by the dashed line in Figure 7) corresponded to a p-value of about 10^-4 ^for W2 and 10^-6 ^for C2, apparently because neglecting day-of-week effects produces spurious cluster alerts when C2 is used. For this 0.02 common background alert rate, we found an empirical detection probability of 0.55 for W2 vs 0.37 for C2. For p-values < 10^-4^, the plots show that C2 and W2 have similar sensitivities, but C2 has a much higher background cluster rate.

**Figure 8:**
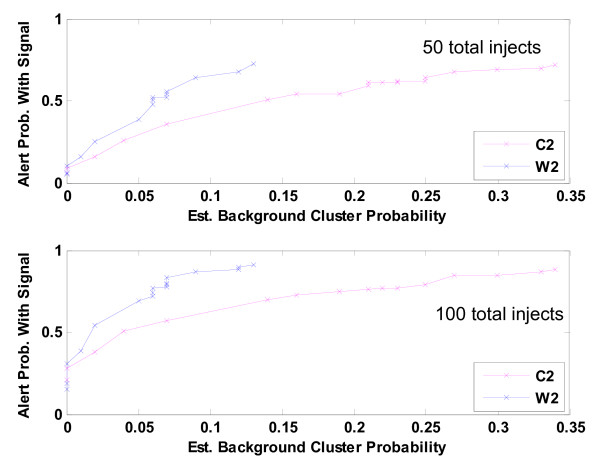
**Receiver Operating Characteristic (ROC) analysis grouped by 50 (a) and 100 (b) injects with either C2 or W2 detection methods**.

In (a) and (b), the x-axis gives the estimated background cluster rates from the dotted curves of Figure 7, and the y-axis gives the estimated signal detection rates from the solid curves. Figure 8(a) was derived for 100 sets of 50 injected counts, while Figure 8(b) is the same for 100 injected counts. Thus, in Figure 8(b) for 100 added cases, the 0.02 background level corresponds to 0.55 and 0.37 for the W2 and C2 estimation methods, as discussed above. For the zip codes of the injected cases, these plots are analogous to ROC curves giving sensitivity as a function of false alarm rate. Thus, for the 50-inject signals, a 5% background alarm rate gives sensitivities of 38% and 28% for cluster detection using the W2 and C2 estimation methods, respectively. For the 100-inject signals, the analogous sensitivity estimates are approximately 70% for W2 and 50% for C2.

## Discussion

The above results substantially support the idea that accurate spatial estimation methods are essential for robust detection of anomalous spatial clusters at controlled background cluster rates. Among the SaTScan user community, when an expected spatial distribution of cases is not available in the form of a population file with reliable estimates, a common method for deriving subregion expected counts is the conditioning on marginal distributions for both dates and subregions, as in the space-time permutation scan statistic [11]. For the purely spatial scan statistic, this conditioning is equivalent to a special case of the moving baseline methods described above, with the test period data included in the baseline and with no buffer between baseline and test cases. The advantage of this method is that there is never a problem of assessing the likelihood of cases in a subregion that has no baseline cases. The disadvantage is that the baseline is biased by the test period cases. Effects of this disadvantage can become significant if the baseline case counts are sparse. The estimation methods in the current paper provide an alternative whereby, in SaTScan terms, the Poisson method could be used and the population file updated with subregion estimates from a moving baseline separated from the test period by a buffer. In this alternative, if case records occur in the test period from subregions that have no baseline cases, expected values are computed without bias by the test cases, as discussed in the Appendix. Given the current baseline interval, the problem is then to estimate an expected spatial distribution that accounts for key factors such as seasonality and day-of-week patterns. The above study compares several estimation methods for two syndrome groupings and two levels of spatial resolution of the Texas DoD data.

The evaluation criteria presented for comparing these estimation methods are the background cluster rate of each method and the ability to detect injected signals. For the background cluster rate, Figures 5 and 6 offer some practical guidance:

a) The complexity of spatial estimation should be appropriate for the time series structure and reflect what is known about the data. Simple methods are preferable unless the data structure is rich enough to derive advantage from more complex methods such as finer stratification (e.g., the DOW method) or the regression modeling of [4]. More complex methods have a greater dependence on data quality and involve expense for development of new data sources and convergence issues (e.g., for nonlinear regression).

b) Choosing the baseline interval length involves a trade-off between stability and capturing recent data behavior. In the charted results for the flat average (C2 based) method, 7-day baseline estimates produced consistently higher background cluster rates than the 28-day estimates. Lengthening the baseline further gives more stability, but can actually degrade the clustering performance if the time series mean changes either because of unmodeled seasonal effects or because of altered data provider participation or other common changes in the data acquisition process. Lengthening the baseline from 28 days to 56 days yielded an advantage for certain situations, such as when the DOW stratification was used, but in some situations produced higher background cluster rates.

c) In some health monitoring systems, a single alerting method with fixed parameters might be required for operational reasons. Given such a requirement, the weekday or weekend/holiday stratified averages (W2-based) with a 28-day baseline provide a safe and sometimes the best choice among the methods tested for the DoD outpatient visit data. The unstratified averages might yield marginally better results if day-of-week effects are absent, but could fare much worse if such effects are present. Moreover, day-of-week effects are not always apparent from visual inspection.

d) The results suggest that more robust clustering performance is possible in a system whereby the estimation method and its parameters could be chosen according to the data. For example, one could use day-of-week stratification time series with rich counts like the respiratory syndrome and large subregions, as in the facility-level runs. A longer baseline could be advantageous for more rare syndrome groupings such as rash, especially if seasonal effects are absent.

The experience of this study also suggested that the p-value threshold for cluster significance should be determined by test runs using authentic data for different outcome variables. Finally, clustering runs on background data could help evaluate the utility of syndrome groupings. The year-round high mean and variance of the respiratory syndrome count data lead to a high background cluster rate, as measured by the sequential background runs. This observation suggests a more selective record filtering, such as a subsyndrome for influenza-like illness. The runs using the sparser rash syndrome counts support this approach as did additional runs with other syndrome groupings.

## Conclusion

This study was conducted as part of an effort to bridge the gap between surveillancerelated research and the need for robust and sensitive routine health monitoring. The BioSense data environment has grown to include various types of data from an increasing number of hospitals and other clinical data providers. Statistical behaviors such as seasonal trends, day-of-week effects, and age distributions vary among these sources, and appropriate syndromic classifications are still under study. Next steps are to seek appropriate spatial estimation methods for the new BioSense clinical data sources and record groupings and to standardize the method selection criteria. Regardless of the cluster detection method of choice in the future, reliable spatial background estimation will remain essential for robust surveillance.

## Competing interests

The authors declare that they have no competing interests.

## Authors' contributions

JX carried out the evaluation of spatial estimation methods and cluster detection studies, participated in the design, and drafted the manuscript.

HB conceived of the study, participated in its design and coordination, carried out the ROC analysis and helped to draft the manuscript.

LM carried out and programmed C source code for the signal simulation and injection studies, and draft the portion of manuscript.

JE carried out and programmed space-time scan statistics study cross-checked in Matlab, implemented and tested Gumbel algorithm in Matlab.

ML carried out and programmed space-time scan statistics study in C.

JT conceived of the study, and participated in its design and helped to draft the manuscript.

All authors read and approved the final manuscript.

## Appendix

Computing expected values for each sub-region by the modified baseline method:

Let *s *be a spatial index, *t *be a temporal index, *D *be a type of day (i.e., Sunday, Monday, Saturday, holiday) and *t(D) *is temporal index associated with type of day. An expected value at geo-coordinate [*s*] on day [*t(D)*] can be given by equation (1),

(1)

Where, *prob*[*s*(*D*)], given by equation (2), is the probability of case found at geo-coordinate [*s*] during the baseline period on certain type of day *D*.

(2)

The *testperiod*[*t*(*D*)], given by equation (3), is the summation of all geo-coordinates cases on test day [*t(D)*].

(3)
